# Surgical Lectures and Essays

**Published:** 1904-12

**Authors:** 


**Surgical Lectures and Essays.** By A. MARMADUKE SHEILD, M.B. Pp. 312. London: The Medical Publishing Company, Limited. [N.D.]

This is an interesting and useful little book. We have not discovered much that is very original in the articles it contains, and most of the chapters are reprinted clinical lectures, and are therefore specially intended for students, but they may also be read with profit by practitioners. As might be expected from Mr. Sheild as the author of a book on diseases of the breast, many chapters are devoted to this subject. Some deal with abdominal surgery; and such subjects as the treatment of fractures, ulcers of the tongue, and goitre are also considered. Mr. Sheild's remarks about the management of fracture are full of practical lessons. He explains why he differs from Mr. Arbuthnot Lane in his recommendation that a very large number of simple fractures should be operated on. The book may be taken up by the busy practitioner and a chapter read in short intervals of leisure or in driving about (for the print is very large and clear), and it will not fail to interest and instruct.

